# The efficacy and safety of extended thromboprophylaxis after colorectal surgery: a systematic review and meta-analysis

**DOI:** 10.1007/s00384-025-05002-9

**Published:** 2025-10-09

**Authors:** Joseph Do Woong Choi, Nguyen Huynh, Talia Shepherd, Aswin Shanmugalingam, Fiona Lee Gavegan, Karen Shedden, Amy Cao, Nimalan Pathmanathan, Toufic El-Khoury, Kerry Hitos, James Wei Tatt Toh

**Affiliations:** 1 Department of Colorectal Surgery, Westmead Hospital. Corner Hawkesbury Road and Darcy Roads, Westmead, NSW Australia; 2 Faculty of Medicine and Health, The University of Sydney, Sydney, NSW Australia; 3 School of Medicine, University of Notre Dame, Sydney, NSW Australia; 4 Westmead Research Centre for Evaluation of Surgical Outcomes, Department of Surgery, Westmead Hospital, Corner Hawkesbury Road and Darcy Roads, Westmead, NSW Australia

**Keywords:** Extended thromboprophylaxis, Low molecular weight heparin, Direct oral anticoagulant, Venous thromboembolism, Bleeding

## Abstract

**Purpose:**

To report (1) efficacy and (2) safety outcomes comparing extended thromboprophylaxis (ETP) with low molecular weight heparin (LMWH) and direct oral anticoagulants (DOAC) versus no ETP after colorectal surgery.

**Methods:**

A systematic review using MEDLINE, EMBASE and Cochrane Central Register of Controlled Trials to March 2024. Randomised controlled trials and observational studies investigating ETP with LMWH or DOAC for at least 28 days were included. (1) Efficacy outcome was venous thromboembolism (VTE) rate at 30-days postoperatively. (2) Safety outcomes included major bleeding events at 30–90-days. Odds ratio (OR) and 95% confidence intervals (CI) were calculated using a Mantel–Haenszel fixed effects model.

**Results:**

Five studies (2936 patients) investigating colorectal surgery for colorectal cancer (CRC) or inflammatory bowel disease (IBD) were included. Total VTE rates were significantly reduced in the LMWH ETP group compared with no ETP [0.56% (OR 0.18, 95% CI 0.07–0.50]. The DOAC ETP group was also compared with no ETP with lower total VTE rates [OR 0.22, 95% CI 0.08–0.67]. There was no difference in VTE rates between LMWH and DOAC ETP. There were no differences in major bleeding events in the LMWH ETP group at 90-days [OR 0.25, 95% CI 0.03–2.24], or the DOAC ETP at 30-days [OR 2.68, 95% CI 0.32–22.35]. There were no VTE-related mortalities at 90-days in either group.

**Conclusion:**

ETP after colorectal surgery for CRC and IBD reduced the incidence of VTEs with no impact on major bleeding within 90-days. ETP for at least 28 days should be considered particularly for those who have a high risk for postoperative VTE.

**Supplementary Information:**

The online version contains supplementary material available at 10.1007/s00384-025-05002-9.

## Introduction

Venous thromboembolism (VTE) is a common cause of early mortality contributing to approximately 50% of post cancer surgery mortality [1, 2]. Patients undergoing colorectal surgery have additional risk factors such as advanced age, limited mobility, need for pelvic dissection and indication for operation (malignancy, inflammatory bowel disease) [3, 4].

A study involving 32,000 patients with colorectal cancer (CRC) and inflammatory bowel disease (IBD) reported a VTE incidence of 2.9%–3.1% at 30-days and 4.3% at 90-days postoperatively [5]. Patients with malignant disease undergoing major surgery have twice the risk of postoperative deep vein thrombosis (DVT) and non-fatal pulmonary embolism (PE) and triple the risk of fatal PE compared to surgery for benign conditions [6, 7]. Additionally, IBD patients are inherently at a higher risk for developing VTE due to the hypercoagulable state of the disease [8].

To reduce the risk of developing VTE, it is standard of care that patients undergoing major colorectal surgery are administered anticoagulation postoperatively as inpatients. However, post hospital discharge, there is significant variation in the utilisation of thromboprophylaxis [9].

A National Surgery Quality Improvement Program (NSQIP) review of over 116,000 patients found that one third of postoperative DVT and PE after colorectal surgery occurred after hospital discharge [10]. The median time to occurrence of VTE after abdominal surgery is 15 days [11]. Following these findings, randomised controlled trials (RCTs) have tested the efficacy and safety of extended thromboprophylaxis (ETP) using either low molecular weight heparin (LMWH) or direct-acting oral anticoagulants (DOACs) [6, 12, 13]. The latest Cochrane review on ETP with LMWH for abdominal or pelvic surgery showed that ETP was associated with an 80% relative risk reduction in postoperative DVT and PE [14]. These developments in the literature have led to numerous guidelines suggesting ETP following colorectal surgery.

National Institute for Health and Care Excellence (NICE) and American College of Chest Physicians (ACCP) guidelines recommend the use of ETP with LMWH for four weeks following major abdominal or pelvic cancer surgery [15, 16]. However, the uptake of ETP has been variable [9, 14, 17]. This may be due to a perceived lack of clinical importance of VTE as an endpoint, as many of the studies report reductions in asymptomatic DVTs without differences in mortality risk [17]. The clinical relevance of asymptomatic distal DVTs and associated risk factors for proximal clot extension are not completely understood. In fact, the latest American Society of Colon and Rectal Surgeons (ASCRS) Clinical Practice Guidelines suggests that in patients undergoing resection for CRC or IBD deemed to be high risk for VTE, ETP *may* be considered, with a conditional recommendation strength [18].

There are emerging RCTs on the use of DOACs as an alternative to LMWHs for ETP in terms of 28-day postoperative VTE rates and bleeding events [19–21]. In fact, compared to standard therapy DOACs had significantly improved participant satisfaction and were more cost-effective in preventing both postoperative DVT and clinically relevant non-major bleeding events [19, 22]. However, these studies were based after gynecologic cancer surgery therefore the generalisability to colorectal surgery is questionable.

Currently, there are no meta-analyses that investigate ETP specific to colorectal surgery, with colorectal guidelines mainly guided from the pooled results from a generalised abdominal and pelvic surgery cohort. The aims of this meta-analysis are to report efficacy and safety outcomes comparing ETP with the use of LMWH and DOACs versus no ETP specifically after colorectal surgery.

## Methods

A systematic review of RCTs and observational studies prescribed extended thromboprophylaxis ≥ 28 days with either LMWH or DOAC following benign or malignant colorectal surgery were examined. The study followed the Preferred Reporting Items for Systematic Reviews and Meta-Analysis (PRISMA) statement. The study was registered in the PROSPERO International Prospective Register of Systematic Reviews (ID number: CRD42024543046).

### Search strategy

A literature search was conducted systematically using MEDLINE via Pubmed, EMBASE and Cochrane Central Register of Controlled Trials (CENTRAL) from inception to March 2024. Medical Subject Heading (MeSH) terms ‘thromboembolism,’ ‘extended prophylaxis’ and ‘colorectal surgery’ were used. Manual screening of abstracts from major haematology and oncology conferences (e.g. American Society of Haematology, European Haematology Association, and American Society of Clinical Oncology), screening of clinical trials registries, references from retrieved studies and grey literature was also conducted (Supplementary 1 and 2).

### Study selection and eligibility criteria

The inclusion criteria were: (1) RCTs and observational studies (including conference abstracts); (2) adult patients ≥ 18 years old; (3) colorectal surgery for benign and malignant indications; (4) directly compared extended LMWH/DOAC thromboprophylaxis with a non-extended (control) thromboprophylaxis group; (5) extended thromboprophylaxis was for ≥ 28 days after index operation; (6) reported efficacy: DVT, PE and/or safety outcomes: bleeding, mortality; (7) use of thromboprophylaxis with LMWH or DOAC after surgery; (8) VTE was diagnosed using compression ultrasound for DVT and computed tomography pulmonary angiography or ventilation-perfusion scan for PE. The study excluded non-colorectal surgery studies, case reports, descriptive articles, animal studies, non-English studies and those not investigating extended thromboprophylaxis. Proximal DVT was defined as thrombosis involving the popliteal vein and above, and distal DVT as isolated calf vein thrombosis. Major bleeding was defined as per the International Society on Thrombosis and Haemostasis [20, 21] or as defined by the investigators.

### Data extraction and quality assessment

Covidence (Veritas Health Innovation Ltd) was used to support citation screening. Three investigators (JC, NH, TS) selected the title, abstract, full text of studies and extracted the data independently. Consensus was reached for any discrepancies, and were reviewed by a fourth investigator (AS).

The quality of the RCTs was assessed by two reviewers (NH and AS) using the Cochrane Risk of Bias 2 (RoB2) tool. Disagreements were reviewed by a third investigator (JC) and consensus was reached by discussion. Observational studies were assessed using the Newcastle–Ottawa scale.

### Outcome measures

The primary efficacy outcomes were radiologically confirmed total VTE, total DVT and proximal or symptomatic DVT within a 30-day postoperative period after colorectal surgery. The primary safety outcomes were major bleeding events, all-cause mortality and VTE-related mortality at 30 to 90-days postoperatively.

### Statistical analysis

Estimated pooled odds ratio (OR) and 95% confidence outcomes were calculated using a Mantel–Haenszel fixed effects model using Review Manager (RevMan), Version 5.3 program. Copenhagen: The Nordic Cochrane Centre, The Cochrane Collaboration, 2014. An *I*^2^ statistic estimated heterogeneity from variation across studies rather than chance, with *I*^2^ > 50% considered significant heterogeneity. All *p*-values were 2-sided, with *p* < 0.05 defining statistical significance. Publication bias was not examined owing to less than ten studies examined.

## Results

### Study selection and characteristics

**Fig. 1:**
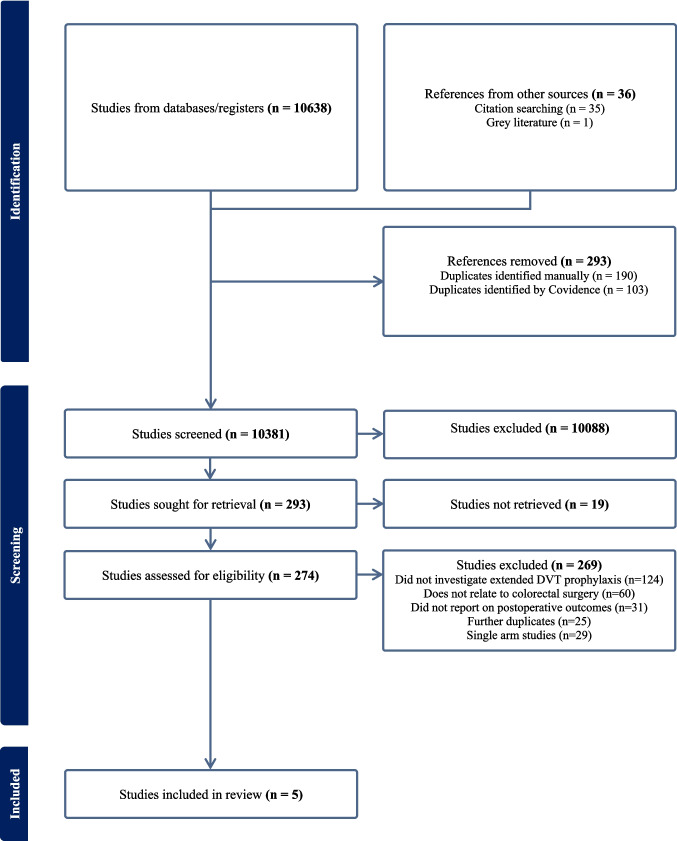
PRISMA flow diagram summarising screening and selection process

A total of 10,674 articles were identified of which 274 were selected for full text reviews to assess for eligibility. A total of 5 studies (3 RCTs and 2 observational studies) comprising 2936 CRC or IBD patients were included (Fig. 1 and Table 1). The proportion of patients who had CRC or IBD were not described in one study [23].

**Table 1:** Summary of studies from systematic review of ETP vs control in colorectal surgery

Study	Design	Arms	Duration of treatment	Number	Age (years)	Male sex (%)	Type of colorectal surgery	Indication for colorectal surgery	Intervention	Outcomes	Follow-up
[12]	RCT	E C	28 ± 2 days 8 ± 2 days	112 113	65 (median) 66 (median)	55 58	Laparoscopic	CRC	Enoxaparin 4000 UI, dalteparin 5000 UI or nadroparin 2850 UI	Total VTE 30 days: - E: 0/112 - C: 11/113 Total proximal or symptomatic DVT 30 days: - E: 0/112 - C: 2/113 Major bleeding 90 days: - E: 0/112 - C: 1/113	4 weeks, 3 months
[13]	RCT	E C	4 weeks 7 ± 2 days	287 282	65.8 (mean) 64.5	56.8 50	Laparoscopic	CRC	Rivaroxaban 10 mg	Total VTE 30 days: - E: 3/287 - C: 11/282 Major bleeding 30 days: - E: 2/287 - C: 0/282	4 weeks, 3 months
[24]	RCT	E C	56 days 3–7 days	307 307	61.4 (mean) 60.8 (mean)	58 61	Open or laparoscopic	CRC	Tinzaparin 4500 IU	Major bleeding 90 days: - E: 0/307 - C: 2/307	56 days
[25]	Observational	E C	30 days Unspecified	121 (VTE cohort), 363 (bleeding cohort) 168 (VTE cohort), 54 (bleeding cohort)	55 (mean) 58 (mean)	44.6 52.4	Open or minimally invasive	CRC or IBD	Rivaroxaban 10 mg	Total VTE 30 days: - E: 1/121 - C: 8/168 Major bleeding 30 days: - E: 4/363 - C: 0/54	30 days
[23]	Observational	E C	28 days Unspecified	605 660	Unspecified Unspecified	Unspecified Unspecified	Unspecified	CRC or IBD	Low molecular weight heparin	Total VTE 30 days: - E: 4/605 - C: 14/660 Total proximal or symptomatic DVT 30 days: - E: 4/605 - C: 14/660	30 days

*RCT* randomised controlled study, *E* experimental arm, *C* control arm, *VTE* venous thromboembolism, *CRC* colorectal cancer, *IBD* inflammatory bowel disease, *DVT* deep vein thrombosis

### Risk of bias and sources of funding

One of the three RCTs had a low risk of bias [13], and two of them had some concerns with bias due to deviations from intended intervention (D2) [12, 24], as per the RoB2 tool (Supplementary 3a-c and 4). This is because although the outcome assessors were blinded, the patients and healthcare providers were not in the latter two RCTs and therefore, there was some risk for bias. In addition, the primary outcome for one of the RCT was disease-free survival at 3 years as a result of intervention, rather than VTE or bleeding risk [24]. One of the observational studies received eight stars (out of nine) from the Newcastle–Ottawa scale suggesting high quality. The other received six stars to indicate fair quality, owing to the limitations of an abstract, and a missing statement about the adequacy of follow-up of the cohort. The full scoring of quality assessment and risk of bias is in Supplementary 5a-b.

One RCT declared funding from Bayer (Italy) who were not involved in the design or conduct of the study [13]. Another RCT was sponsored by the various Canadian hospitals and research organisations, The Cancer Research Society and Leo Pharma without any involvement in the design or conduct of the study [24]. The third RCT received sponsorship from the University of Perugia but declared that it was a no-profit study [12], and the two observational studies had no or unknown funding declarations.

### Efficacy outcomes

#### All venous thromboembolic events

**Fig. 2:**
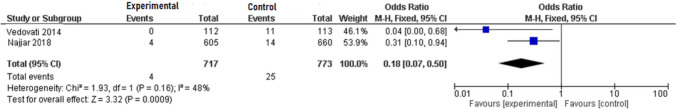
Forest plot of comparison: LMWH ETP vs no ETP, outcome: Total VTE 30 days

**Fig. 3:**
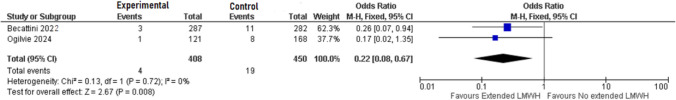
Forest plot of comparison: Rivaroxaban ETP vs no ETP, outcome: Total VTE 30 days

**Fig. 4:**
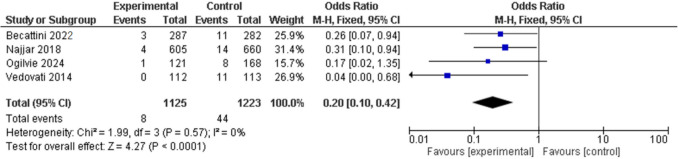
Forest plot of comparison: LMWH and Rivaroxaban ETP vs no ETP, outcome: Total VTE 30 days

The incidence of total VTE events at 30 days for LMWH ETP was extracted from one RCT and one observational study. The incidence of total VTE was significantly reduced in the LMWH ETP group compared to no ETP [0.56% (4/717) vs. 3.23% (25/773); OR 0.18, 95% CI 0.07–0.50] (Fig. 2). The incidence of total VTE events at 30 days for rivaroxaban ETP was also extracted from one RCT and one observational study. Similarly, the incidence of total VTE events was significantly lower in the rivaroxaban ETP group compared to no ETP [0.98% (4/408) vs. 4.22% (19/450); OR 0.22, 95% CI 0.08–0.67] (Fig. 3). When LMWH and rivaroxaban ETP data were pooled, there was significantly reduced total VTE events in the ETP group compared to the control group at 30 days [0.71% (8/1125) vs. 3.6% (44/1223); OR 0.2, 95% CI 0.10–0.42] (Fig. 4).

#### Proximal or symptomatic DVT events

**Fig. 5:**
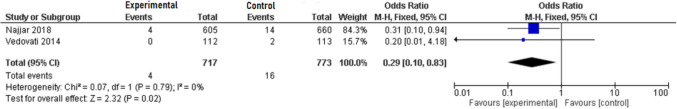
Forest plot of comparison: LMWH ETP vs no ETP, outcome: Total proximal or symptomatic DVT 30 days

The incidence at 30 days was significantly reduced in the LMWH ETP group compared to no ETP [0.56% (4/717) vs 2.07% (16/773); OR 0.29, 95% CI 0.10–0.83] (Fig. 5).

#### Total DVT events (proximal, distal, symptomatic, asymptomatic)

**Fig. 6:**
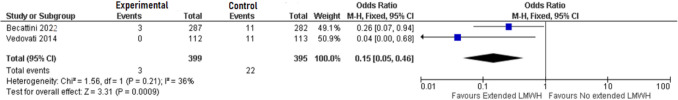
Forest plot of comparison: LMWH and Rivaroxaban ETP vs no ETP, outcome: Total DVT 30 days

The incidence at 30 days was significantly reduced at 30 days when LMWH and rivaroxaban ETP were analysed together [0.75% (3/399) vs. 5.57% (22/395); OR 0.15, 95% CI 0.05–0.46] (Fig. 6).

### Safety outcomes

#### Major bleeding events

**Fig. 7:**
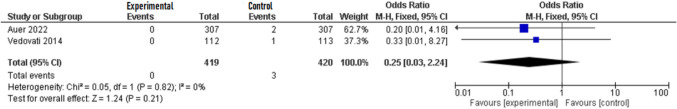
Forest plot of comparison: LMWH ETP vs no ETP, outcome: Major bleeding 90 days

**Fig. 8:**
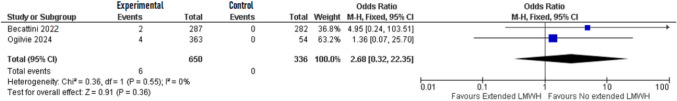
Forest plot of comparison: Rivaroxaban ETP vs no ETP, outcome: Major bleeding 30 days

**Fig. 9:**
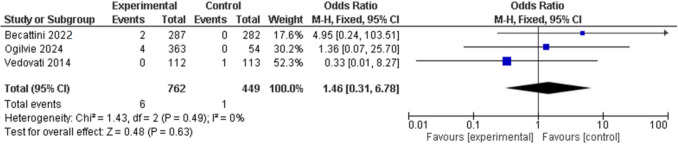
Forest plot of comparison: LMWH and Rivaroxaban ETP vs no ETP, outcome: Major bleeding 30 days

There was no statistical differences in major bleeding events in the LMWH ETP group compared to the control group at 90 days [0% (0/419) vs 0.71% (3/420); OR 0.25, 95% CI 0.03–2.24] (Fig. 7). This result was also seen with rivaroxaban ETP analysis at 30 days [0.92% (6/650) vs 0% (3/336); OR 2.68, 95% CI 0.32–22.35] (Fig. 8) and when LMWH and rivaroxaban ETP data were pooled at 30 days [0.79% (6/762) vs 0.22% (1/449); OR 1.46, 95% CI 0.31–6.78] (Fig. 9).

### Mortality events

There were no reported VTE-related mortality events in any of the five studies. There were three all-cause mortality events within 90-days; one patient randomised to LMWH ETP died from pancreatitis, and two patients who were both from control arms died from cancer progression.

## Discussion

The results of this study demonstrated that in patients undergoing colorectal surgery for CRC or IBD, there was a significant reduction of total VTE events, proximal or symptomatic DVT events and total DVT events with the use of ETP with either LMWH or DOAC (specifically rivaroxaban) for at least 28 days post-operatively.

No differences in major bleeding events with the use of LMWH or rivaroxaban ETP were observed compared to the control group at 30 or 90 days. The rates of major bleeding events were minimal, accounting for < 1% in either group. Only two of the studies reported VTE-related mortality, both of which did not report any deaths in either the ETP or control group at 90 days [12, 13]. There were two deaths that were unrelated to VTE in one study at 90 days, precipitated by pancreatitis in one patient and cancer progression in another [12]. The study suggests a favourable risk-to-benefit assessment for ETP, possibly supporting the wider implementation and utilisation of ETP with either LMWH or DOAC after colorectal surgery.

These results were demonstrated in many other RCTs, systematic reviews, and meta-analysis [6, 14, 18, 19, 25, 26], which were largely based on a heterogeneous group of patients that broadly underwent surgery for abdominal, pelvic, and/or gynaecology oncology surgery for a variety of indications. Guidelines for thromboprophylaxis for colorectal surgery are based on pooled results from a range of major abdominal surgery, not just colorectal surgery [15, 16]. To date, there is only one published guideline on the use of ETP specifically after colorectal surgery. The ASCRS Clinical Practice Guidelines (2023) have a conditional strength of recommendation that ETP *may* be considered in patients undergoing resection for CRC or IBD if deemed to be at high risk for VTE [17]. However, this guideline did not detail the type of thromboprophylaxis to be used, the exact duration of ETP or the specific high-risk features for VTE development; however, it did acknowledge that the duration of ETP has not yet been determined due to the paucity of studies directly comparing the duration of ETP [17].

Regarding high-risk features, the Caprini score has been validated as a risk prediction tool in observational studies to predict VTE after colorectal surgery [27–30]. In a prospective study, the highest VTE risk in screened VTE patients represented a Caprini score of ≥ 12 (40.5%) or between 9 and 12 (20.4%) after laparoscopic colorectal surgery [27]. There are recommendations to suggest the use of 30 days of thromboprophylaxis in Caprini score ≥ 9 [31]; however, this tool is not always used. There are currently no high-quality studies that have investigated ETP after colorectal surgery for non-IBD benign pathology to suggest any thromboprophylaxis recommendations.

The current literature included in this meta-analysis needs to be interpreted cautiously because the studies mostly cited asymptomatic, screen-detected DVT, with two studies demonstrating no VTE-related mortality in the ETP or control group at 90 days. The relevance of asymptomatic DVT has been questioned, with the majority confined to the lower extremity veins with limited embolisation potential [14]. Unresolved questions include the proportion of asymptomatic screen-detected DVTs that resolve without intervention or progress to clinically relevant VTE, and the risk of bleeding with the prescription of anticoagulation for asymptomatic DVTs. However, asymptomatic VTE may have clinical importance beyond a ‘surrogate’ endpoint. There are reports of 59% increased relative risk of post-thrombotic syndrome, and a significant association between proximal VTE and 90-day mortality with asymptomatic DVT [32, 33]. Patients after orthopaedic surgery given LMWH have demonstrated a relative reduction in asymptomatic DVT translating into a reduction in symptomatic DVT [34–36]. Another aspect about the current literature is the relatively small (and some zero) efficacy events in both the experimental and control groups, with wide CI. Although VTE events may be statistically significant in the experimental group, comparison with small absolute event differences with the control group may be difficult to interpret, and to base recommendations in clinical practice.

There are several limitations to consider. First, there is heterogeneity of the population analysed from five studies, including pathology (CRC and IBD) and operative technique (open and minimally invasive). The number of CRC and IBD patients were not described in one study, and it is difficult to understand the relative VTE risk contributions between these two pathologies [22]. Secondly there were differences in the control arms, with one study describing an undefined period of short heparin withdrawal after 8 ± 2 days after surgery [12], another ceasing inpatient LMWH after 7 ± 2 days [13], and other studies stopping thromboprophylaxis after a variable or unknown number of inpatient days [22, 23, 37]. In addition, there may have been a selection bias in one RCT [12] as randomisation was undertaken a few days after surgery. Thus, patients who tolerated LMWH during the first week after surgery with no adverse events were more likely to be included in the study and some heparin induced adverse events may not have been captured. There was also selection bias in one observational study where they selected patients with a Caprini score of ≥ 5 [22]. The LMWH regimens differed within and between studies or were not specified [12, 22, 23]. Furthermore, there may have been event-related bias from single investigator-blinded studies in two RCTs [12, 23]. Also, in two observational studies, there was no mention of whether symptomatic or asymptomatic DVT or PE were accounted for to report a total VTE rate [22, 37]. Finally, the current applicability of ETP within an Enhanced Recovery After Surgery (ERAS) protocol in colorectal surgery, where there is an emphasis on early mobilisation and early discharge may not necessarily be translated from study results that predated ERAS protocols.

## Conclusion

The results of this study suggest the efficacy and safety of LMWH or DOAC ETP use after colorectal surgery for CRC and IBD. The rate of DVT and PE was significantly lower with the use of at least 28 days of LMWH or DOAC (rivaroxaban), attributed mainly from asymptomatic DVT events. The bleeding rates were low and similar between LMWH and DOAC ETP.

Although these results are promising, the data needs to be interpreted cautiously at present, as there are currently insufficient RCTs to make definite recommendations. Based on this study, the authors suggest ETP with LMWH for at least 28 days in select patients who have high risk for VTE after colorectal surgery for CRC or IBD. There are greater efficacy and safety data with the use of LMWH than DOACs at present, as are the current guidelines for ETP after abdominal surgery based on LMWH [15, 16]. ETP should be prescribed on an individualised basis using a VTE risk assessment model that considers the risk/benefit ratio.

## Supplementary Information

Below is the link to the electronic supplementary material.

Supplementary file 1 (DOCX 85.3 KB)

## Data Availability

No datasets were generated or analysed during the current study.
